# Low adherence to cardiovascular risk assessment guidelines in patients with rheumatoid arthritis: a retrospective chart review of routine clinical practice

**DOI:** 10.1007/s00296-025-05916-1

**Published:** 2025-06-26

**Authors:** Louise Murphy, Grainne Murphy, Nicola Cornally, Sheena McHugh, Mohamad M. Saab, Patrick Cotter

**Affiliations:** 1 Catherine McAuley School of Nursing and Midwifery, University College Cork, Cork, T12 AK54 Ireland; 2 Department of Rheumatology, Cork University Hospital, Wilton, Cork, T12 DC4A Ireland; 3 School of Public Health, University College Cork, Cork, T12 XF62 Ireland

**Keywords:** Cardiovascular diseases, Patient care, Practice guidelines as topic, Retrospective studies, Rheumatoid arthritis, Risk assessment

## Abstract

**Introduction:**

Patients with rheumatoid arthritis (RA) have an elevated risk of developing cardiovascular disease (CVD). Despite European guidelines recommending routine CVD risk assessment, implementation in clinical practice is challenging. The objectives of this review were to determine if patients attending an Irish tertiary rheumatology centre received CVD risk assessments in line with European guidelines and assess the extent of CVD risk factor screening over five years of routine rheumatology care.

**Methods:**

A retrospective chart review was conducted for patients newly diagnosed with RA in 2018, with five-year follow-up. Data were extracted to determine if CVD risk assessments were performed, and where absent, risk was retrospectively calculated. The Strengthening the Reporting of Observational Studies in Epidemiology (STROBE) statement checklist for cohort studies was used to guide the reporting of this study.

**Results:**

Among 21 patients, no documented CVD risk assessments were identified. CVD risk factor screening was consistently insufficient. There was a lack of documented clinical data necessary to conduct a CVD risk assessment on more than half of patients at study entry, and one quarter of patients at five-year follow up. Of those with data available (n = 10), retrospective calculations showed 80% had an undetected moderate or higher CVD risk at diagnosis. There was no documented referral to primary care for CVD risk assessment.

**Conclusion:**

Implementation of CVD risk management guidelines in the routine care of patients with RA is challenging. The interpretation and operationalisation of guideline recommendations by rheumatology healthcare professionals in relation to implementation barriers needs to be explored.

**Supplementary Information:**

The online version contains supplementary material available at 10.1007/s00296-025-05916-1.

## Introduction

Patients with rheumatoid arthritis (RA) have an increased risk of developing cardiovascular disease (CVD), resulting in heart failure, myocardial infarction, and stroke [1, 2]. Meta-analyses of European and North American population-based studies undertaken from 1960 to 2011 indicated a 48% increased risk of CVD in patients with RA, with a 50–60% increased risk of CVD-related mortality compared to the general population [3–5]. More recent retrospective cohort studies from the United States of America [6], Europe [7], Canada [8], and China [9] have demonstrated a steady decline in the incidence of cardiovascular events in patients with RA, mirroring the decline seen in the general population since the late 1990s. Although global CVD mortality rates in patients with RA have also declined in the past 20 years [10–13], a substantial mortality gap remains between patients with RA and the general population [13–16].

European recommendations for CVD risk management in patients with RA have been published by the European Alliance of Associations for Rheumatology (EULAR) [17]. These recommendations indicate that it is the treating rheumatologist’s responsibility to ensure all patients with RA have a CVD risk assessment performed once every five years and when there is a major change in rheumatic medications (i.e., initiation or switching of any biological agent [17] or targeted synthetic disease modifying anti-rheumatic drug [18]). EULAR recommendations also state that CVD risk assessment should be undertaken according to individual countries’ national guidelines where available. In countries where national guidelines are not available, the Systemic Coronary Risk Evaluation (SCORE) CVD risk prediction tool should be used to calculate CVD risk with the addition of a 1.5 multiplication factor [17]. EULAR recommendations also suggest that rheumatologists should document who is performing CVD risk assessments for patients with RA and must ensure each patient is aware of the need for routine CVD risk assessment.

Suboptimal rates of CVD risk assessment and CVD risk factor screening (necessary to conduct CVD risk assessments) have been reported in clinical practice [19–21]. A recent scoping review found a lack of robust evidence detailing the CVD risk assessment practices of rheumatology healthcare professionals (HCPs) in the routine care of patients with RA [22]. Further research is required to understand the current CVD risk assessment practices of rheumatology HCPs in the clinical setting. Therefore, the aim of this retrospective chart review was to explore if patients with RA attending the rheumatology service of a large university teaching hospital and tertiary referral centre in the south of Ireland are being CVD risk assessed as part of routine care in line with European guidelines. The objectives were:

i. To ascertain if a CVD risk assessment was undertaken at least once in five years of routine rheumatology care and after each major change in rheumatology treatments including the initiation of medications that increase CVD risk. Where CVD risk assessment was not performed, to retrospectively calculate patients CVD risk at study entry and at five-year follow-up.
ii. To identify what CVD risk assessment tools were used by rheumatology HCPs use in routine care.
iii. To summarise the extent of CVD risk factor screening documented by rheumatology HCPs over five years of routine rheumatology care.

## Methods

### Design

An observational cohort study was conducted by undertaking a retrospective chart review. The Strengthening the Reporting of Observational Studies in Epidemiology (STROBE) statement checklist for cohort studies (Online Resource 1) was used to guide the reporting of this study [23].

### Setting

Medical records were sought for all patients with a new diagnosis of RA, attending the rheumatology service of a large university hospital and tertiary referral centre located in the south of Ireland. The rheumatology centre serves a catchment population of 584,156 [24] and operates under the governance structure of a model 4 university teaching hospital. In Ireland model 4 hospitals are tertiary referral centres that provide a wide range of sub-specialties delivering ambulatory and inpatient care with an Emergency Department on site [25]. Ethical approval was granted by the Clinical Research Ethics Committee of the Cork Teaching Hospitals (protocol reference ECM 4 (L) dated July 30th, 2024) with wavier of informed consent.

### Participants

As European recommendations were published in 2017 [17], we included all patients newly diagnosed with RA in the following year (between 1st January and 31st December 2018) regardless of disease severity. Given that these recommendations suggest CVD risk assessment should be undertaken for all patients with RA at least once every 5 years, the decision was made to examine patients’ routine care over a five-year period, from 2018 to 2023. To reduce selection bias, a non-probability purposive sample of medical records of all adult patients aged 18 years and above, seen for their first time by a rheumatology HCP in 2018 with a documented diagnosis of serology positive or serology negative RA, were included. The process of making a clinical diagnosis of RA is the prerogative of an experienced rheumatologist and is often distinct from that used in disease classification criteria [26]. Therefore, in line with work published in this area [27–29], we included medical records of patients who had a diagnosis of RA made solely from the clinical assessment of a rheumatologist, as opposed to meeting international RA classification criteria [30].

We excluded medical records of patients diagnosed with juvenile arthritis or any other form of pediatric inflammatory joint disease and patients younger than 18 years of age. Patients with a known history of CVD at the time of diagnosis were excluded, as the presence of CVD requires immediate clinical management. CVD status was confirmed by reviewing patients’ medical records, including referral letters, medical and nursing notes, and correspondence from GPs.

### Variables

#### Main outcome variable

The primary outcome was documented CVD risk assessment (yes/no), indicating whether rheumatology HCPs conducted CVD risk assessment for patients with RA at least once in five years of routine care and after any major change in anti-rheumatic treatment, in line with current guidelines [17].

#### Quantitative and other variables

Variables that were measured at baseline in 2018 included patient demographics (age and gender) and symptom duration in months. As the guideline refers to the cautionary use of corticosteroids and non-steroidal anti-inflammatory drugs (NSAIDs) in relation to impact on CVD risk, we sought documented evidence of cumulative dosing for both medications [17]. As there has been controversy in recent years over targeted synthetic disease modifying antirheumatic medications increasing CVD risk in certain cohorts [31–35] we also collected data in relation to the number of times patients were commenced on one of these agents. Data were collected on CVD risk factors addressed in the SCORE2 [36] and the SCORE2-OP [37] risk calculators: hypertension, dyslipidaemia, and smoking.

Hypertension was defined as a blood pressure ≥ 140/90 mmHg [38], a high body mass index (BMI) was defined as ≥ 25 kg/m^2^ [39], an elevated (fasting) blood sugar was defined as any result above 100 mg/dl (5.6 mmol/L). An elevated HbA1c of ≥ 42 mmol/mol was deemed relevant to screen for diabetes risk. Dyslipidaemia was defined as a serum elevation of one or more of the following lipid parameters; low-density lipoprotein (LDL), total cholesterol, or triglyceride levels, or a reduced high-density lipoprotein (HDL) cholesterol level [40]. Smoking was defined as smoking cigarettes or using e-cigarette vaping with fluid containing nicotine and/ or tar. An ex-smoker was defined as a patient who had not smoked in the past six months [41]. The presence of CVD risk factors was recorded as ‘yes’ when there was documented evidence in the chart of the diagnosis or when there was documented evidence of individual CVD risk factor medication management. We also examined whether obesity and diabetes mellitus screening was carried out as outlined in Keeling et al.’s retrospective chart review of CVD risk factor assessment in patients with inflammatory arthritis and lupus [42]. We identified rheumatology HCPs as being any professional with the responsibility of caring for patients with rheumatic conditions, who was directly aligned with the hospital’s rheumatology department at the time of the review. This included physicians, nurses, physiotherapists, and occupational therapists.

### Procedures

A review code book including variable definitions was designed to aid data extraction (Online Resource 2). The data extraction tool was piloted by (LM) and (GM) independently on four patients with a new diagnosis of RA in 2017. Data from the pilot were not included in the data analysis.

#### Study size

The medical record numbers of all new and return adult attendees to the rheumatology department in 2018 were collected from all rheumatology outpatient clinic lists between 1st January 2018 to 31st December 2018. Every record number was exported to a Microsoft Excel sheet and duplicates were removed. The remaining medical record number for each patient was used to search the hospital’s file-share system used to store all typed correspondence letters in 2018. Patients who received a new diagnosis of RA in 2018 or during any subsequent visit and who met the inclusion criteria were identified for enrolment.

#### Data sources and measurement

Data collection was undertaken using researcher-developed raw and coded data extraction forms guided by the aim and objectives of this study. The first rheumatology outpatient attendance or in-patient consultation after 1st January 2018 was used as the baseline visit and the medical records were reviewed for a full five years of routine care. If patients had no scheduled outpatient appointment at the five-year follow-up point, the following clinic visit by any rheumatology HCP in 2023 was deemed their exit point.

Handwritten medical records, typed and digital medical correspondence, and laboratory reports of eligible patients were examined. Only personal data obtained by the hospital for the purposes of providing routine healthcare to the patient were accessed. Patient data were de-identified and reported in aggregate in-line with ethical standards.

Each patient’s CVD risk assessment was retrospectively calculated by applying the SCORE2 risk assessment tool [36] to patients aged 40–69 years and the SCORE2-OP [37] tool to patients aged 70–89 years. The calculations were based on clinical data from the time closest to the baseline visit at study entry point in 2018 and repeated using clinical data closest to the study exit point five years later in 2023. For patients who were younger than 40 years of age who had sufficient clinical data, the QRisk3 tool [43] was used to estimate CVD risk. Clinical data within six months of study baseline and exit were used for retrospective calculation. In keeping with European guidelines [17], the 1.5 multiplication factor was added to all retrospective SCORE calculations to account for RA related CVD risk. As the QRisk3 calculator includes RA as an independent risk factor, application of the 1.5 multiplication factor was not required [43]. CVD risk was classified as low, moderate, high, or very high. Patients with high or very high risk had a letter sent to their General Practitioner (GP) (i.e., Family Doctor) requesting an updated risk assessment in primary care. Data extraction for the main study was undertaken by (LM) from 9th September to 23rd December 2024.

### Statistical methods

Data analysis was undertaken using descriptive statistics. The Shapiro–Wilk test was used to check normality of all metric data. Continuous variables were reported using means and standard deviations for data with a normal distribution. Medians and interquartile ranges were used to report non-normally distributed data. Categorical data were reported using percentages and frequency counts, with percentages rounded to one decimal place. Categories with zero values were omitted from tables.

## Results

### Participants and descriptive data

**Fig. 1:**
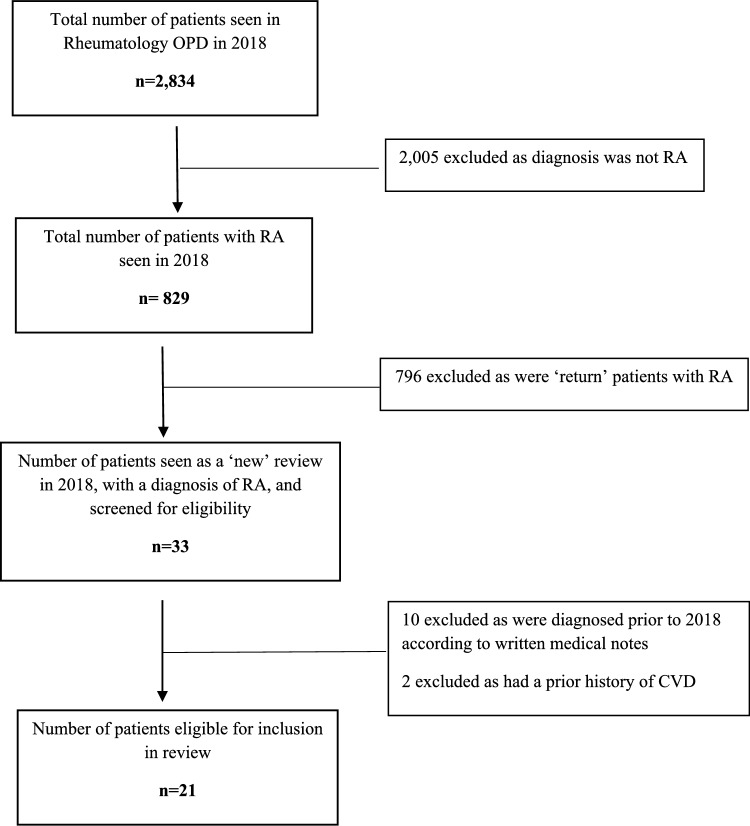
Flow Diagram for Eligibility Screening

In 2018, the rheumatology outpatient department saw a total of 2,834 individual adult patients. A review of all 2,834 GP correspondence letters for eligibility showed 829 patients seen in 2018 had a diagnosis of RA, of which, 33 were new to the service. Of those, 21 were deemed eligible for inclusion in this study (Fig. 1).

Patient characteristics, clinical healthcare utilisation, and clinical data representing baseline patient group and subsection analysis of those at follow-up who completed five years of routine care are shown in Table 1. The mean (SD) age of patients in 2018 at baseline was 53.5 (12.7) years. Over half of participants were female (57.1%, n = 12). The median symptom duration at baseline was seven months (IQR: 3.5, 15 months). Of the 21 patients included, five (23.8%) did not complete five full years of routine care due to discharge from service (9.5%, n = 2), patient death (9.5%, n = 2), and lost to follow-up (4.8%, n = 1).

**Table 1:** Patient characteristics, clinical healthcare utilisation, and clinical data representing baseline patient group and subsection analysis of those at follow-up who completed five years of routine care

Characteristic	Baseline (n = 21)	Follow-up (n = 16)
Age at baseline (years)^a^	53.5 (12.7)	52.8 (11)
Gender^b^		
Female	12 (57.1)	9 (56.3)
Male	9 (42.9)	7 (43.8)
Symptom duration at baseline (months)^c^	7 (3.5, 15)	6.5 (3.5, 18)
Consultations per patient^c^		
Face-to-face	11 (6, 14.5)	12 (9.5, 17.5)
Virtual	1 (0.5, 1)	1 (1, 1.5)
Change in Rheumatic Treatment		
Any major change^b^		
Never	9 (42.9)	5 (31.3)
1–2 times	6 (28.6)	5 (31.3)
3–4 times	4 (19.1)	4 (25)
5 + times	2 (9.5)	2 (12.5)
Central tendency/ variability	1 (0, 3)^c^	2.06 (1.94)^a^
Commenced on JAK-I^b^		
Never	18 (85.7)	13 (81.3)
Once	3 (14.3)	3 (18.8)
Central tendency/ variability	0 (0)^a^	0 (0)^a^
≥ 30 days of ≥ 5mg corticosteroid^b^		
Never	15 (71.4)	10 (62.5)
1–2 times	4 (19.1)	4 (25)
3–4 times	2 (9.5)	2 (12.5)
Central tendency/variability	0 (0, 1)^c^	0 (0, 1)^c^
≥ 3 days/week of NSAID ≥ 1 year^b^		
Never	21 (100)	16 (100)
Hypertension		
Blood pressure documented at:^b^		
50- 60% of visits	2 (9.5)	0 (0)
61–80% of visits	2 (9.5)	1 (6.3)
81–100% of visits	17 (81)	15 (93.8)
Elevated blood pressure documented^b^		
Never	4 (19.1)	2 (12.5)
1–2 times	6 (28.6)	4 (25)
3–4 times	6 (28.6)	6 (37.5)
5 + times	5 (23.8)	4 (25)
Raised body mass index		
Height documented at:^b^		
0% of visits	19 (90.5)	14 (87.5)
1–10% of visits	2 (9.5)	2 (12.5)
Weight documented at:^b^		
0% of visits	1 (4.8)	0 (0)
50–60% of visits	1 (4.8)	0 (0)
61–80% of visits	4 (19.1)	4 (25)
81–100% of visits	15 (71.4)	12 (75)
Body mass index calculated at:^b^		
0% of visits	19 (90.5)	14 (87.5)
1–10% of visits	1 (4.8)	1 (6.3)
11–20% of visits	1 (4.8)	1 (6.3)
Elevated body mass index documented^b^		
Never	19 (90.5)	14 (87.5)
1 time	2 (9.5)	2 (12.5)
Diabetes mellitus		
HbA1c/Blood glucose documented at:^b^		
0% of visits	20 (95.2)	15 (93.8)
1–10% of visits	1 (4.8)	1 (6.3)
Elevated HbA1C/Blood glucose on laboratory results^b^		
Never	16 (76.2)	11 (68.8)
1–2 times	1 (4.8)	1 (6.3)
3–4 times	3 (14.3)	3 (18.8)
5 + times	1 (4.8)	1 (6.3)
Dyslipidaemia		
Lipid profile documented at:^b^		
0% of visits	14 (66.7)	9 (56.3)
1–10% of visits	2 (9.5)	2 (12.5)
10–20% of visits	3 (14.3)	3 (18.8)
20–30% of visits	2 (9.5)	2 (12.5)
Dyslipidaemia reported on laboratory results^b^		
Never	6 (28.6)	3 (18.8)
1–2 times	4 (19.1)	3 (18.8)
3–4 times	4 (19.1)	4 (25)
5 + times	7 (33.3)	6 (37.5)
Smoking		
Smoking status documented at:^b^		
0% of visits	2 (9.5)	1 (6.3)
1–10% of visits	5 (23.8)	5 (31.3)
11–20% of visits	7 (33.3)	5 (31.3)
21–30% of visits	3 (14.3)	2 (12.5)
31- 40% of visits	2 (9.5)	2 (12.5)
41- 60% of visits	2 (9.5)	1 (6.3)
Smoking status (baseline/ subsequent visit)^b^		
Not documented	3 (14.3)	2 (12.5)
Non-smoker	8 (38.1)	5 (31.3)
Ex-smoker	4 (19.1)	3 (18.8)
Current smoker	6 (28.6)	6 (37.5)

*JAK-I*, Janus Kinase inhibitor; *NSAID*, Non-steroidal anti-inflammatory drug

^a^Mean (standard deviation)

^b^n (%)

^c^Median (interquartile range)

**Fig. 2:**
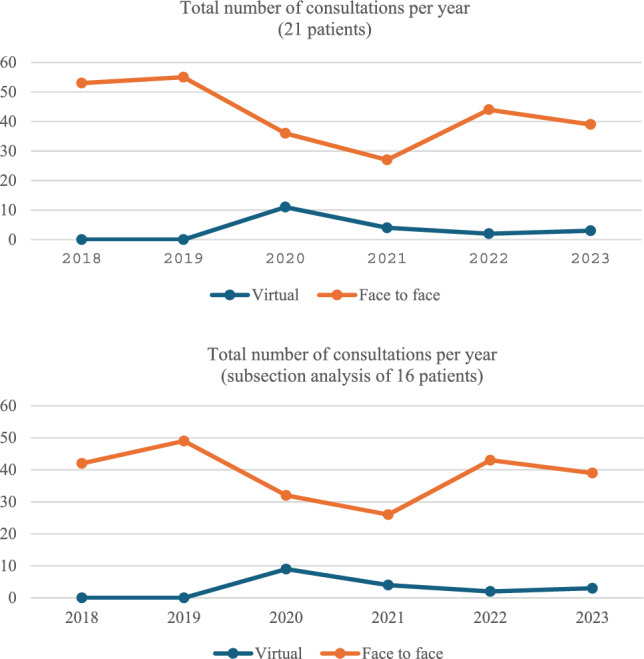
Total number and type of patient consultations per year of study

The number of face-to-face and virtual consultations varied each year, reflecting the impact of COVID-19. In 2020, virtual consultations increased, coinciding with a decline in face-to-face visits (Fig. 2). The trend demonstrating the rise in virtual and decline in face-to-face consultations for the 21 patients included at baseline was mirrored in the subsection analysis of yearly consultation type reflecting the 16 patients who completed five years of routine care. The difference between both groups was mostly seen in the years 2018 and 2019 indicating that the five patients lost to follow-up contributed most to the face-to-face consultation numbers for those years (Fig. 2).

### Outcome data and main results

#### Cardiovascular disease risk assessment

None of the 21 patients had a CVD risk assessment completed by any rheumatology HCP at any time point over the five-year study period. Hence, there was no documented evidence of any CVD risk assessment tool used in practice. There was documented evidence of lifestyle and CVD risk factor education delivered by a rheumatology HCP in the medical notes of five (23.8%) patients.

#### Retrospective calculation of cardiovascular disease risk

In 2018, at study entry, 52.4% (n = 11) of patients had insufficient clinical or laboratory data to retrospectively calculate CVD risk. Of the 10 patients with data available, 20% (n = 2) had a low, 30% (n = 3) had a moderate, 20% (n = 2) had a high, and 30% (n = 3) had a very high risk of developing CVD. In 2023, of the 16 patients who completed five years of follow-up care, 25% (n = 4) had insufficient clinical or laboratory data to retrospectively calculate CVD risk. Of those with laboratory data available, 16.7% (n = 2) had a low, 33.3% (n = 4) had a moderate, 16.7% (n = 2) had a high, and 33.3% (n = 4) had a very high risk of developing CVD.

#### Cardiovascular disease risk progression from baseline to five-year follow-up

Subsection analysis of those who completed five full years of routine rheumatology care demonstrated CVD risk progression or reduction from baseline to five-year follow-up could not be identified in 50% (n = 8) of patients due to insufficient data at one or both time points. A little over one-third (37.5%, n = 6) of patients were calculated to have the same risk stratification at baseline compared to five-year follow-up, with one patient (6.3%) scoring low, two scoring moderate (12.5%), one scoring high (6.3%), and the other two patients (12.5%) scoring very high risk at both time points. Just one patient (6.3%) had a reduction in CVD risk from a high to moderate level. In contrast, one patient (6.3%) had an increase in CVD risk stratification from a low to moderate level from baseline to follow-up (Table 2).

**Table 2:** Comparison of retrospective calculation of patients’ CVD risk stratification at baseline and at five-year follow-up (n = 16)

Patient	Baseline	Change	Follow-up
(a)	Moderate	↔	Moderate
(b)	Moderate	↔	Moderate
(c)	Low	↔	Low
(d)	Low	↑	Moderate
(e)	Unable to assess	–	Very high
(f)	Unable to assess	–	Very high
(g)	Unable to assess	–	Unable to assess
(h)	Unable to assess	–	Unable to assess
(i)	Unable to assess	–	Unable to assess
(j)	Very high	↔	Very high
(k)	Very high	↔	Very high
(l)	High	↔	High
(m)	High	↓	Moderate
(n)	Moderate	–	Unable to assess
(o)	Unable to assess	–	High
(p)	Unable to assess	–	Low

↔ No change in risk factor stratification

↑ Increase in cardiovascular risk

↓ Reduction in cardiovascular risk

– Unable to compare data

#### Episodes of care where cardiovascular disease risk assessment should have been considered

For all patients included at baseline, 57.1% (n = 12) had more than one major change in rheumatic treatment, with some patients (9.5%, n = 2) having more than five changes in rheumatic medication over five years of routine care. Changes in medication included commencement of a Janus Kinase inhibitor, an agent known to increase the risk of thromboembolic events [44] which was prescribed in 14.3% (n = 3) of patients. Corticosteroid was documented at a rate of greater than 30 days use of 5 mg or higher in 28.6% (n = 6) of patients. No patient had a documented use of NSAIDs for more than three days a week beyond one year (Table 1). When major changes in anti-rheumatic treatment are factored into account, there were 34 additional episodes of care in the medical notes of 12 patients where rheumatology HCPs should have been prompted to undertake a CVD risk assessment. Furthermore, of these 12 patients, five had a documented use of more than 5 mg corticosteroid daily for longer than 30 consecutive days, which due to their impact on CVD risk, should have lowered the HCP’s threshold for conducting a CVD risk assessment. Evidence of patient education regarding RA related CVD risk, lifestyle, or CVD risk factor advice (smoking cessation, weight management, dietary advice, exercise advice) from a rheumatology HCP was only seen in 23.8% (n = 5) of patients’ medical notes.

#### Cardiovascular disease risk factor: hypertension

Blood pressure was documented at 88.9% of all face-to-face out-patient clinic visits (n = 176 blood pressure recordings over 198 outpatient department [OPD] visits). Of the 21 patients included at baseline, 17 (81%) had at least one episode of elevated blood pressure at a rheumatology clinic between 2018 and 2023; however, only one (4.8%) had intervention from a rheumatology HCP in the form of weight management advice to address hypertension. There was no documented evidence of any recommendation from rheumatology to patients’ GPs to have a CVD risk assessment undertaken in primary care in any of these 17 cases. One patient (4.8%), however, was referred to their GP to address hypertension as an individual risk factor, and two patients (9.5%) had evidence of antihypertensive treatment at their subsequent clinic visits (Table 3).

**Table 3:** Documentation and communication of cardiovascular disease risk factor management to patients General Practitioner

Indicator^a^	Hypertension	Raised body mass index	Raised HbA1C/Blood glucose	Dyslipidaemia	Smoking
No. of patients with CVD RF documented in medical notes by rheumatology HCP	17	2	1	7	6
No. of patients with CVD RF (evident on retrospective review of laboratory results)	–	–	5	15	–
No. of patients with documented intervention by rheumatology HCP to treat/ manage individual risk factor	1	1	1	0	4
No. of patients with recommendation from rheumatology HCP to GP to address as independent risk factor	1	0	0	2	0
No. of patients with recommendation from rheumatology HCP to GP to do CVD risk assessment in primary care	0	0	0	0	0
No. of patients with evidence of CVD risk factor management noted by rheumatology at subsequent clinic visits	6^b^	1^c^	0	1^d^	1^e^

*HbA1C* glycated haemoglobin; *No* number; *CVD* cardiovascular disease; *RF* risk factor; *HCP* healthcare professional; *GP* General Practitioner

^a^At any time point over five years of routine care

^b^Use of antihypertensive noted in two patients, intentional weight loss noted in 4 patients

^c^Engagement in exercise

^d^Use of lipid lowering agent

^e^Use of nicotine replacement therapy

#### Cardiovascular disease risk factor: high body mass index

Weight was documented in 89.4% (n = 177 of 198) of all face-to-face out-patient clinic visits, with height only documented in 1% (n = 2 of 198). Rheumatology HCPs documented a raised BMI for 9.5% (n = 2) of 21 patients who had both weight and height measured. BMI was calculated in one patient (4.8%) once, while one other patient (4.8%) had a BMI calculated twice. Only one of these patients (4.8%) received lifestyle advice from a rheumatology HCP. No documented recommendation was made to either of patients’ GPs to address high BMI as an individual risk factor.

#### Cardiovascular disease risk factor: diabetes mellitus

Of the 21 patients, 23.8% (n = 5) had elevated glycated haemoglobin or glucose levels available to view on the laboratory system, with one patient (4.8%) demonstrating a raised glycated haemoglobin or glucose on 11 separate occasions. Rheumatology HCPs documented one episode of raised glycated haemoglobin and gave subsequent lifestyle advice. None of the patients’ medical records had a documented recommendation to the GP to address diabetes mellitus as an independent risk factor for CVD.

#### Cardiovascular disease risk factor: dyslipidaemia

Lipoprotein results were noted by rheumatology HCPs in one-third of patients’ medical notes (n = 7, 33.3%), reflecting 14 individual episodes of dyslipidaemia documented by rheumatology HCPs over five years. Of these seven patients, 71.4% (n = 5) had improved lipid levels at the following clinic visit. No intervention was undertaken by a rheumatology HCP on any patient; however, two patients had a recommendation to their GP to address dyslipidaemia as an independent risk factor, while another had a documented use of a lipid lowering agent (Table 3). A review of laboratory results for all 21 patients over the five-year period demonstrated dyslipidaemia was reported by the hospital laboratory 92 times on 15 (71.4%) patients over five years of routine care. None of the 21 patients had any documented evidence of a GP referral to a cardiologist or any written record of cardiology review in their medical notes over the five-year study period.

#### Cardiovascular disease risk factor: smoking

Smoking status was documented for 85.7% (n = 18) of the 21 patients at baseline, of which 33.3% were current smokers (n = 6), 22.2% were ex-smokers (n = 4), and 44.4% had never smoked (n = 8). Lifestyle advice was given to two-thirds of current smokers (n = 4, 66.7%) by a rheumatology HCP, and a smoking cessation referral to a hospital smoking cessation officer was sent by a rheumatology HCP for one patient (16.7%). None of the six patients (33.3%) who smoked had a referral from a rheumatology HCP to their GP to address smoking cessation in primary care. One patient (16.7%) had evidence of risk factor management in the form of nicotine replacement therapy and 66.7% of current smokers (n = 4) at baseline had documented evidence by rheumatology of cessation attempts, 50% (n = 2) being successful at five-year follow-up.

## Discussion

### Summary of findings

Despite European guidelines recommending CVD risk assessment every five years and after major rheumatic treatment changes, our findings indicate that, in a single large rheumatology centre, these assessments are not routinely documented as part of routine care. CVD risk factor screening was consistently insufficient, resulting in a lack of documented clinical data needed for risk assessment in over half of patients at study entry and a quarter of patients at the five-year follow-up.

Of those who had clinical data documented, retrospective calculation of CVD risk demonstrated 80% of patients had an undetected moderate to very high CVD risk at time of diagnosis. At five-year follow-up, of those that had clinical data documented, 83.3% had an undetected moderate to very high CVD risk. The most consistently screened CVD risk factor was hypertension, followed by smoking status at baseline. Patients weight measurements were frequently documented at face-to-face clinic visits; however, height (necessary to calculate BMI) was only documented on one occasion for two patients. Communication to patients’ GPs about CVD risk factor assessment was inadequate, with only three documented recommendations for assessment in primary care over five years of routine care.

### Lack of guideline implementation

It has been reported that, despite being aware of RA related CVD risk, rheumatologists do not systematically screen for risk factors in practice [45]; this finding is consistent with our study. Barriers to CVD risk factor screening and subsequent assessment in rheumatology clinics (e.g., time, human resources, a lack of standardised protocols, and the asymptomatic nature of risk factors) have been identified [45–48]. Another reason may be that rheumatology HCPs prioritise control of RA disease activity over targeting a reduction in overall CVD risk. It is well understood that elevations in acute phase reactants, pro-inflammatory cytokines, and circulating RA specific autoantibodies in states of high RA disease activity increases endothelial dysfunction and promotes atherosclerotic plaque formation [49–51]. Therefore, it is not surprising that a reduction in systemic inflammation mitigates CVD risk in patients with RA [49, 52, 53]. While targeting RA disease control to reduce systemic inflammation and subsequent CVD risk is crucial, this approach alone is insufficient, as it overlooks the impact of traditional risk factors such as hypertension, smoking, and dyslipidaemia on CVD risk. Adequate identification of these traditional CVD risk factors is necessary so HCPs can tailor preventative care strategies and advise patients on lifestyle modifications to reduce future cardiovascular risk. Medications used to treat RA such as corticosteroids and NSAIDs, and in certain cohorts JAK inhibitors, can also increase CVD risk resulting in hypertension, thrombus formation and an increase in insulin resistance [54–57]. For this reason, rheumatology HCPs must balance disease control with cardiovascular safety in patients with RA, which includes the recognition of preventative care and the importance of a formal CVD risk assessment in practice.

### Cardiovascular disease risk factor screening

In our review, 75% (n = 12) of 16 patients had sufficient CVD risk factor screening data documented to facilitate retrospective calculation of CVD risk at five-year follow-up. This rate was higher than previously published chart reviews of only 0.68% (n = 3) [42] and 10% of 107 medical records [20]. Improvement in CVD risk screening documentation seen at five-year follow up in our study may be because of an improved awareness of the importance of CVD risk factor screening and documentation amongst rheumatology HCPs in more recent years compared to 2018. It may also be due to a perception from rheumatology HCPs that CVD risk increases the longer one is diagnosed, perhaps as disease duration was a criterion included in the preceding guidelines [58]. However, RA disease duration does not independently affect CVD risk [59]. For this reason, patients who are newly diagnosed (even those pre-diagnosis) who have high levels of inflammation can be at higher CVD risk [60, 61]. Although an improvement in documented CVD risk screening was observed in this review it is important to note that elements of patient education and/or lifestyle advice that might have been given to patients during the clinic consultation, may not have been documented in the medical notes. As this review relied on documented evidence to report outcomes, we were unable to verify undocumented interventions.

Several CVD risk factor screening and assessment clinic protocols have been reported in the literature with varying degrees of success. Rheumatology nurse-led CVD risk factor screening clinics in the USA reported successful outcomes targeting improved GP follow-up for rheumatic patients with hypertension and those who smoke [62, 63]. A more recent Danish exploratory study reported patients with RA found CVD risk factor screening consultations delivered by trained rheumatology nurses improved their awareness of CVD risk and highlighted the importance of maintaining a healthy lifestyle [64]. A Dutch study reporting results of a dedicated CVD risk screening programme noted that despite targeted advice and systematic referral to patients GPs, only one third of patients who had a very high CVD risk had adjustment of cardio preventative medication [65]. They went on to report that undergoing the CVD risk screening programme prompted a reasonable number of patients to increase their exercise, improve their diet, and lose weight [65]. In comparison a Danish cohort study reported GP follow-up rates of only 50% in patients with RA after receiving CVD risk screening consultation from a trained rheumatology nurse [66]. The authors suggested this may be due to lack of patient awareness regarding the implications of high-risk stratification. This highlights the importance of improving patient education strategies irrespective of the HCP tasked to deliver the intervention and demonstrates that cardiovascular preventative care for patients with RA remains a challenge in routine care.

### Undetected cardiovascular disease risk

We identified several missed opportunities for CVD risk assessment. Every patient consultation is an opportunity for intervention. Missed assessments delay preventative care for high-risk patients. Raising rheumatology HCP awareness of their roles in CVD prevention aligns with the “Making Every Contact Count” initiative [67]. This programme offers HCPs support in helping patients to make better lifestyle choices and lower their risk of chronic disease as is endorsed by the Health Service Executive, Ireland’s publicly funded healthcare system [68].

Disease severity assessments in real-world settings often rely on clinical opinion rather than validated composite measures [69]; therefore, the perception that a patient may not have a high CVD risk due to a low frequency of CVD risk factors may have impacted the rheumatology HCPs decision to undertake a formal risk assessment in our study. Patients who have extra articular manifestation or autoantibodies have an increased CVD risk [53, 70, 71]. For this reason, rheumatology HCPs may decide that a patient has a certain level of risk attributable to these RA disease specific factors alone and therefore feel that despite evidence to the contrary [72], a formal CVD risk assessment using a composite measure is not required.

### Communication

We found no documented evidence of any communication from a rheumatology HCP to patients’ GPs to have a CVD risk assessment performed in primary care. Research conducted among GPs has reported their willingness to manage, or co-manage, RA-related comorbidities including CVD [45, 73]. European centres in the Netherlands [74] and Denmark [75] have developed shared-care pathways between rheumatologists and GPs to better communicate risk to patients and their GPs. Dedicated cardiology/ rheumatology clinics [76] have also been developed to better target and monitor at-risk patients; however, implementing such clinics worldwide may be problematic due to country-specific reimbursement regulations and differing healthcare policies [77]. Furthermore, even with a dedicated CVD risk screening programme, targeted patient advice and timely communication to GPs, initiating preventative care can still be problematic despite adequate communication to patients themselves and their general physicians about RA-related CVD risk [65]. Our findings report care provided by rheumatology HCPs who work in a publicly funded hospital within a partially integrated healthcare system. In this context, routine monitoring of blood profiles by GPs is not undertaken on all patients with RA. While in other countries collaborative care between rheumatologists and cardiologists may be common practice, it is not routinely implemented in Ireland. We examined all patients’ medical records seeking evidence of cardiology referrals or collaboration, of which we found none.

CVD risk progression, or lack thereof, may be due to interventions already undertaken by GPs as part of primary care management. For example, patients who were hypertensive or who had hypercholesterolaemia early in their disease may have had antihypertensive or lipid lowering medications commenced by their GPs as part of standard primary care management without communication to patients’ rheumatology team. In our sample, evidence of lipid lowering medications were noted in just one of 15 patients who had laboratory evidence of dyslipidaemia, with antihypertensive medications noted in use in two of 17 patients with hypertension. Our findings correspond with other studies reporting poor communication of CVD risk factors between rheumatology HCPs and GPs in the routine care of patients with RA [20, 45, 78].

A major aspect of CVD risk assessment in patients with RA is medication management including patient use of medications known to increase CVD risk. The duration of corticosteroid and NSAID use may not be adequately reflected in written or typed medical notes. Rheumatology HCPs often document current daily use without noting duration of these medications. This becomes relevant when there is prolonged use of either medication resulting in cumulative CVD risk to the patient. GPs often prescribe corticosteroids and NSAIDs in primary care to treat disease flares without consultation with the patent’s rheumatologists, and often for reasons other than control of RA. Improved collaboration between primary care and the rheumatology team would also allow for a more thorough understanding of patients’ use of both corticosteroids and NSAIDs and subsequent impact on CVD risk.

The findings from our study add to the real-world evidence highlighting issues with CVD risk guideline implementation in the routine care of patients with RA. While other papers report the presence of subclinical atherosclerosis in patients with RA and the value of diagnostic imaging techniques in advanced risk stratification [79, 80], we found a complete lack of basic guideline-recommended CVD risk assessment in this patient cohort. Ausserwinkler et al. [81] call for a pragmatic approach to CVD risk management in patients with RA which entails patient education about CVD risk, treating to a target of disease remission, minimal use of corticosteroid, and initiation of a statin where indicated. We believe, this can be achieved by implementing EULAR guidelines into practice. As our review highlights non-adherence to these guidelines in a routine rheumatology care setting, research needs to be undertaken to gain an understanding of the barriers to CVD risk assessment and guideline implementation in the care of patients with RA.

### Limitations

Because this was a retrospective study, data collection was limited to what was documented in patients’ medical records. As is standard in RCRs, our methodology focused on recorded interventions. It is possible that lifestyle advice provided by rheumatology HCPs was not documented and, therefore, not captured in our analysis. In the absence of documented evidence, such interventions could not be included in the dataset.

The small sample size of 21 patients and recruitment from a single centre limit the generalisability of our findings to broader populations or other healthcare settings. However, this review provides evidence of adherence to guidelines which is currently lacking in literature. The primary aim of this study was to identify patterns in treatment that may warrant further investigation. To reduce sampling bias and enhance the relevance of our findings to real-world practice, we included all patients who received a new diagnosis of RA in 2018, allowing us to reflect their routine care over a five-year period. Notably, other studies in the rheumatology literature have used retrospective designs with similar sample sizes to explore routine care and have yielded valuable insights [82–84]. While our findings may be transferable to other centres and are relevant to both rheumatology HCPs, organisational leaders, and service managers, we advise caution in interpreting our results given the limited sample size.

## Conclusion

Findings and lessons learned from our study indicate that routine CVD risk assessment and screening for patients with RA must be integrated into routine care to align with European guidelines. A standardised shared care protocol between rheumatology HCPs and GPs could improve screening, communication, and timely assessments; however, practical implementation of shared care protocols in routine care may also be inconsistent in certain healthcare contexts.

Future research is needed to explore how best to implement CVD risk assessment into the routine care of patients with RA to align with European recommendations. In a partially integrated healthcare system like Ireland’s, effective implementation of clinical guidelines may require a multifactorial approach. This could involve upgrades to hospital structure and functional systems, revisions to GP referral criteria into rheumatology services, and the development of integrated, multidisciplinary clinics for CVD risk screening and assessment in patients with RA. Understanding the real-world barriers and facilitators to the practical implementation of European guidelines in the context of healthcare delivery is critical for the successful and sustainable implementation of these recommendations into routine care. Exploratory research is needed to understand how rheumatology HCPs perceive, interpret, and apply European guidelines in the routine care of patients with RA. Such research should also explore their views on the individual, systemic, and organisational factors that facilitate or hinder guideline implementation.

## Supplementary Information

Below is the link to the electronic supplementary material.

Supplementary file1 (DOCX 24 KB)

Supplementary file2 (DOCX 80 KB)

## Data Availability

The data underpinning this retrospective chart review consist of sensitive patient information and are therefore not publicly available. De-identified aggregate datasets may be made available by the corresponding author upon reasonable request and subject to appropriate institutional and ethical approvals.
