# Supplementary material for: Low adherence to cardiovascular risk assessment guidelines in patients with rheumatoid arthritis: a retrospective chart review of routine clinical practice
Source: Rheumatol Int. 2025 Jun 26;45(7):158. doi: 10.1007/s00296-025-05916-1 (PMC12202671; doi:10.1007/s00296-025-05916-1)
Supplement: Supplementary file 1 — Supplementary file1 (DOCX 24 KB) [file 296_2025_5916_MOESM1_ESM.docx]

**Online Resource 1:** The RECORD statement – checklist of items, extended from the STROBE statement, that should be reported in observational studies using routinely collected health data.

| **The RECORD statement checklist, extended from the STROBE statement** | | | |
| --- | --- | --- | --- |
| Item | No | Description | Location in manuscript |
| Title and Abstract | | | |
|  | 1.1 | The type of data used should be named in the title or abstract. Where possible, the names of the databases used should be included. | Page 1 |
|  | 1.2 | If applicable, the geographic region and time frame within which the study took place should be reported in the title or abstract. | Page 1 |
|  | 1.3 | If linkage between databases was conducted for the study, this should be clearly stated in the title or abstract. | NA |
| Introduction | | | |
| Background | 2 | Explain the scientific background and rationale for the investigation being reported | Page 2 |
| Objectives | 3 | State specific objectives, including any prespecified hypotheses | Page 3 |
| Methods | | | |
| Study Design | 4 | Present key elements of study design early in the paper | Page 3 |
| Setting | 5 | Describe the setting, locations, and relevant dates, including periods of recruitment, exposure, follow-up, and data collection | Page 3 |
| Patients | 6.1 | The methods of study population selection (such as codes or algorithms used to identify subjects) should be listed in detail. If this is not possible, an explanation should be provided. | Page 3 |
|  | 6.2 | Any validation studies of the codes or algorithms used to select the population should be referenced. If validation was conducted for this study and not published elsewhere, detailed methods and results should be provided. | Page 4 |
|  | 6.3 | If the study involved linkage of databases, consider use of a flow diagram or other graphical display to demonstrate the data linkage process, including the number of individuals with linked data at each stage. | NA |
| Variables | 7 | A complete list of codes and algorithms used to classify exposures, outcomes, confounders, and effect modifiers should be provided. If these codes or algorithms cannot be reported, an explanation should be provided. | Page 4 |
| Data sources/ measurement | 8 | For each variable of interest, give sources of data and details of methods of assessment (measurement). Describe comparability of assessment methods if there is more than one group. | Page 6 |
| Bias | 9 | Describe any efforts to address potential sources of bias | Page 3 |
| Study size | 10 | Explain how the study size was arrived at. | Page 3 |
| Quantitative variables | 11 | Explain how quantitative variables were handled in the analysis. If applicable, describe which groupings were chosen, and why. | Page 6 |
| Statistical methods | 12 | Describe all statistical methods, including those used to control for confounding.  Describe any methods used to examine subgroups and interactions. Explain how missing data were addressed.  Cohort study: If applicable, explain how loss to follow-up was addressed.  Describe any sensitivity analysis | Page 6    Page 8  NA    Page 7  NA |
| Data access and cleaning methods | 12.1 | Authors should describe the extent to which the investigators had access to the database used to create the study population. | Page 5 |
|  | 12.2 | Authors should provide information on the data cleaning methods used in the study. | Page 5 |
|  | 12.3 | State whether the study included person-level, institutional-level, or other data linkage across two or more databases. Linkage techniques and methods used to evaluate linkage quality should be provided. | NA |
| Results | | | |
| Patients | 13 | Describe in detail the selection of the persons included in the study (i.e., study population selection), including filtering based on data quality, data availability and linkage. The selection of included persons can be described in the text and/or by means of the study flow diagram. | Page 6, Fig 1 |
| Descriptive data | 14 | Give characteristics of study patients (demographic, clinical, social) and information on exposures and potential confounders | Page 7 |
|  |  | Indicate the number of patients with missing data for each variable of interest | NA |
|  |  | Cohort study: summarise follow-up time (average and total amount) | Page 7-10 |
| Outcome data | 15 | Cohort study: Report numbers of outcome events or summary measures over time | Page 7-10, Table 1 |
| Main results | 16 | Give unadjusted estimates and if applicable, confounder adjusted estimates and their precision (e.g. 95% confidence interval). Make clear which confounders were adjusted for and why they were included | NA |
|  |  | Report category boundaries when continuous variables were categorised. | Page 6 |
|  |  | If relevant, consider translating estimates of relative risk into absolute risk for a meaningful time period | NA |
| Other analyses | 17 | Report other analyses done- e.g. analyses of subgroups and interactions, and sensitivity analyses | Page 7 |
| Discussion | | | |
| Key results | 18 | Summarise key results with reference to study objectives | Page 10 |
| Limitations | 19 | Discuss the implications of using data that were not created or collected to answer the specific research question(s). Include discussion of misclassification bias, unmeasured confounding, missing data, and changing eligibility over time, as they pertain to the study being reported. | Page 14 |
| Interpretation | 20 | Give a cautious overall interpretation of results, considering objectives, limitations, multiplicity of analyses, results from similar studies, and other relevant evidence | Page 14 |
| Generalisability | 21 | Discuss the generalisability (external validity) of the study results | Page 14 |
| Other Information | | | |
| Funding | 22 | Give the source of funding and the role of the funders for the present study | Page 15 |
| Accessibility of protocol, raw data, and programming code | 23 | Authors should provide information on how to access any supplemental information such as the study protocol, raw data, or programming code. | Page 15 |
