# Supplementary material for: Low adherence to cardiovascular risk assessment guidelines in patients with rheumatoid arthritis: a retrospective chart review of routine clinical practice
Source: Rheumatol Int. 2025 Jun 26;45(7):158. doi: 10.1007/s00296-025-05916-1 (PMC12202671; doi:10.1007/s00296-025-05916-1)
Supplement: Supplementary file 2 — Supplementary file2 (DOCX 80 KB) [file 296_2025_5916_MOESM2_ESM.docx]

**Online Resource 2:** Review Code Book

**Retrospective Chart Review Code Book**

**Patient profile**

Q1: Age

Q2: Gender 1=Male (M) 2=Female (F) 3=Non-binary (NB)

Q3: Symptom duration (in months) at first contact with rheumatology in 2018

Q4: Number of rheumatology outpatient attendances (either virtual or face-to-face) from Jan 2018 to Dec 2023

**CVD risk assessment data**

Q5: Documented evidence of a complete CVD risk assessment in patients’ medical notes from Jan 2018 to Dec 2023

1= No

2= Yes

Q6: Number of times the patient had a documented CVD risk assessment from Jan 2018 to Dec 2023

Q7: CVD risk assessment result 2018 at time of initial presentation to rheumatology based on SCORE risk chart with application of 1.5 multiplication factor (Agca et al. 2017).

1= Low

2= Medium

3= High

4= Very High

Q8: CVD risk assessment result 2023 at study exit point based on SCORE risk chart with application of 1.5 multiplication factor (Agca et al. 2017).

1= Low

2= Medium

3= High

4= Very High

Q9: Number of times patient had a major change in rheumatic treatment (i.e. the initiation of a new or switch of a biologic or targeted synthetic disease modifying anti-rheumatic drug (DMARD), after ≥ 30 consecutive days of ≥ 5mgs corticosteroid use per day, or after ≥ 3 days a week of non-steroidal anti-inflammatory drug use for ≥ 1 year).

Q10: Number of times patient was commenced on a JAK inhibitor from 2018 to 2023

Q11: Number of times patient had a documented use of ≥ 30 consecutive days of ≥ 5mgs corticosteroid use per day

Q12: Number of times patient had a documented use of ≥ 3 days a week of non-steroidal anti-inflammatory drug use for ≥ 1 year

Q13: If CVD risk assessment was undertaken at any time point who performed it (or if multiple CVD risk assessments performed who performed the initial assessment within the study period).

1= Consultant

2= Non consultant hospital doctor

3= Nurse

4= Physiotherapist

5= Occupational Therapist

6= Pharmacist

7= Dietitian

8= Vascular Technician

9= General Practitioner

10= Administration personnel

Q14: Where a CVD risk assessment was documented, what composite measure was used (where multiple measures were used what was the initial measure used within the study period).

1= ERS-RA

2= QRISK

3= SCORE

4= JBS score

5= RRS

6= FRS

7= ATTACC-RA

8= PROCAM

9= ASCVD risk estimator plus

10= ACC/AHA

11= CUORE

12= ASSIGN

13= GVRS

14= WHO/ISH

15= DECODE

16= CAREMA

17= JBSRC

18= Other general population measure

19= Other RA disease specific measure

20= No composite measure used

Q15: Documented evidence of any patient education or information given to patients on CVD lifestyle or risk factor advice (smoking cessation, weight management, dietary advice, exercise advice) by any healthcare professional regarding RA related CVD risk at any stage between Jan 2018 to Dec 2023

1= No

2= Yes

Q16: Where CVD risk assessment was undertaken by means of a composite measure, and risk was deemed moderate/ high/ very high what following intervention(s) was/were undertaken by rheumatology. Select all that apply.

1= None

2= New CVD medication commenced to address CVD risk

3= Smoking cessation referral

4= Physiotherapy referral for exercise regime

5= Patient education regarding RA related CVD risk

6= Lifestyle advice

7= Dietary advice

8= Weight management advice

Q17: Where CVD risk assessment was undertaken, and risk was deemed moderate/ high/ very high is there evidence of any recommendation by rheumatology to the patients GP or to cardiology for CVD preventative intervention?

1= No

2= Yes

Q18: Where CVD risk assessment was undertaken, and risk was deemed moderate/ high/ very high, at subsequent clinic visit what documented evidence was there of CVD risk factor management? Select all that apply.

1= None

2= Antihypertensive agent commenced

3= Cholesterol lowering agent commenced

4= Smoking cessation or engagement with a smoking cessation programme

5= Exercise engagement

**CVD risk factor- Hypertension**

Q19: Number of times Blood Pressure documented at rheumatology OPD over 5 years

Q20: Documented evidence of hypertension at rheumatology clinic visit (hypertension is defined by the ESC/ESH guidelines as BP greater than 140 systolic and/or 90 diastolic mmHg)

1= No

2= Yes

Q21: If hypertension was recorded at the clinic visit what of the following intervention(s) was/were actioned by rheumatology at the clinic visit? Select all that apply.

1= None

2= New medication commenced

3= Smoking Cessation Referral

4= Physiotherapy referral for exercise regime

5= Patient education regarding RA related CVD risk

6= Lifestyle advice

7= Dietary advice

8= Weight management advice

Q22: If hypertension was recorded, was there documented evidence of a recommendation by a rheumatology HCP to the patients GP to have a CVD risk assessment in primary care?

1= No

2= Yes

Q23: If hypertension was recorded, was there documented evidence of a recommendation by a rheumatology health care professional to the patients GP to address individual CVD risk factors?

1= No

2= Yes

Q24: If hypertension was recorded, at subsequent OPD visit, was there documented evidence of CVD risk factor management? Select all that apply.

1= None

2= New medication commenced to address CVD risk

3= Smoking Cessation

4= Physiotherapy engagement

5= Patient education regarding RA related CVD risk

6= Lifestyle Advice

7= Dietary advice

8= Weight management advice

**CVD risk factor- high BMI**

Q25: Number of times Body Mass Index documented at rheumatology OPD over 5 years

Q26: Evidence of high BMI recorded by rheumatology anytime over the five years (BMI of ≥ 25)

1= No

2= Yes

Q27: If high BMI was recorded at clinic visit, was there evidence of intervention by any health professional? Select all that apply.

1= None

2= New weight loss Rx

3= Referral to Dietitian for dietary advice

4= Physiotherapy referral for exercise regime

5= Patient education regarding RA related CVD risk

6= Lifestyle advice

7= Dietary advice

8= Weight management advice

Q28: If high BMI was recorded at clinic visit, was there documented evidence of a recommendation by a rheumatology health care professional to the patients GP to have a CVD risk assessment in primary care?

1= No

2= Yes

Q29: If high BMI was recorded at clinic visit, was there a recommendation by a rheumatology health care professional to the patients GP to address individual CVD risk factors?

1= No

2= Yes

Q30: If high BMI was recorded at clinic visit, at subsequent clinic visit was there evidence of BMI related CVD risk factor management? Select all that apply.

1= None

2= Yes, decreased BMI measurement

3= Yes, decreased weight

4= Yes, evidence of improved diet

5= Yes, exercise engagement

**CVD risk factor- Diabetes Mellitus**

Q31: Number of times blood sugar or HbA1c documented at rheumatology OPD over 5 years

Q32: Evidence of elevated blood sugar (fasting) and/ or elevated HbA1c documented by a rheumatology HCP at any stage in five years (≥ 100 mg/dl or 5.6 mmol/L) (HbA1c ≥ 42 mmol/mol).

1= No

2= Yes

Q33: If elevated blood sugar or HbA1c was noted at rheumatology clinic, what of the following intervention(s) were undertaken by rheumatology. Select all that apply.

1= None

2= Referral to endocrinology

3= Referral to nutrition and dietetics

4= Patient education regarding RA related CVD risk

5= Lifestyle advice

6= Dietary advice

7= Weight management advice

Q34: If elevated blood sugar or HbA1c was documented by rheumatology, was there a recommendation by a rheumatology health care professional to the patients GP to address individual CVD risk factors?

1= No

2= Yes

Q35: If elevated blood sugar or HbA1c was recorded at rheumatology clinic, at the subsequent clinic visit, was there evidence of CVD risk factor management regarding diabetes risk? Select all that apply.

1= None

2= Referred to endocrinology by GP

2= Seen by endocrinology

3= New diabetic medication commenced (Metformin, DPP-4 inhibitor, GLP-1 receptor agonist, GLP-1/GIP receptor agonist, SGLT2 inhibitor, Sulfonylurea agent, TZD agent)

4= Referred to dietician by GP

Q36: If elevated blood sugar or HbA1c recorded by rheumatology, at next clinic visit was there evidence of reduced levels?

1= No

2= Yes

**CVD risk factor- dyslipidaemia**

Q37: Number of times hospital standard lipid profile (LDL, HDL, TC, and triglycerides) documented at rheumatology OPD over 5 years

Q38: Evidence of dyslipidaemia documented by rheumatology, or by any other HCP, anytime over the five years (defined as a serum elevation of one or more of the following lipid parameters; low-density lipoprotein (LDL), total cholesterol, or triglyceride levels, or a reduced high-density lipoprotein (HDL) cholesterol level [40].

1= No

2= Yes

Q39: If dyslipidaemia was recorded at a rheumatology clinic, what of the following intervention(s) were undertaken by rheumatology. Select all that apply.

1= None

2= Referral to cardiology

3= Referral to nutrition and dietetics

4= Patient education regarding RA related CVD risk

5= Lifestyle advice

6= Dietary advice

7= Weight management advice

Q40: If dyslipidaemia was recorded was there a recommendation by a rheumatology health care professional to the patients GP to address individual CVD risk factors?

1= No

2= Yes

Q41: If dyslipidaemia was recorded at rheumatology clinic, at the subsequent clinic visit was there evidence of CVD risk factor management? Select all that apply

1= None

2= Referred to cardiology by GP

2= Seen by cardiology

3= Referred to nutrition and dietetics

4= Seen by nutrition and dietetics

5= New medication commenced (statin/ Cholesterol absorption inhibitor/ PCSK9 inhibitor / Adenosine triphosphate lyase (ACL) inhibitors / Bile acid sequestrant / Fibrates

Q42: If hyperlipidemia recorded by rheumatology, at next clinic visit was there evidence of reduced cholesterol levels?

1= No

2= Yes

**CVD risk factor- Smoking**

Q43: Number of times smoking status documented at rheumatology OPD over 5 years

Q44: Smoking status at study entry point in 2018 (or when first documented in notes after study entry point)

1= Non-smoker (Never smoked)

2= Current smoker (including social smoking/ someday or any day smoking)

3= Ex-smoker (self-reported complete smoking cessation for ≥ 6 months)

Q45: If smoking was noted at rheumatology clinic, was there intervention by rheumatology. Select all that apply.

1= None

2= Referral to smoking cessation specialist

3= Referral to pharmacist to discuss nicotine replacement therapy

4= Patient education regarding RA related CVD risk

5= Lifestyle advice

6= Request to GP to explore smoking cessation options

Q46: If smoking was recorded was there a recommendation by a rheumatology health care professional to the patients GP to address individual CVD risk factors?

1= No

2= Yes

Q47: If smoking was recorded at rheumatology clinic, at the subsequent clinic visit was there evidence of CVD risk factor management? Select all that apply.

1= None

2= Seen by smoking cessation specialist

3= Smoking cessation addressed by GP

4= Patient using or has used nicotine replacement therapy or a nicotinic receptor agonist (gum/ transdermal patch/ nasal spray/ oral inhaler/ oral tablets/ Varenicline)

Q48: If smoking was recorded by rheumatology, at subsequent clinic visit was there written evidence of cessation attempts documented by rheumatology?

1= No

2= Yes but unsuccessful

3= Yes and patient is ≤ 6 months off cigarettes

4= Yes and patient is ≥ 6 months off cigarettes
